# Arrhythmogenic right ventricular cardiomyopathy in patients with biallelic *JUP*-associated skin fragility

**DOI:** 10.1038/s41598-020-78344-9

**Published:** 2020-12-10

**Authors:** Hassan Vahidnezhad, Leila Youssefian, Masoomeh Faghankhani, Nikoo Mozafari, Amir Hossein Saeidian, Fatemeh Niaziorimi, Fahimeh Abdollahimajd, Soheila Sotoudeh, Fateme Rajabi, Liaosadat Mirsafaei, Zahra Alizadeh Sani, Lu Liu, Alyson Guy, Sirous Zeinali, Ariana Kariminejad, Reginald T. Ho, John A. McGrath, Jouni Uitto

**Affiliations:** 1 Jefferson Institute of Molecular Medicine, Thomas Jefferson University, Philadelphia, PA USA; 2 Department of Dermatology and Cutaneous Biology, Sidney Kimmel Medical College, Thomas Jefferson University, 233 S. 10th Street, Suite 450 BLSB, Philadelphia, PA 19107 USA; 3 Molecular Medicine Department, Biotechnology Research Center, Pasteur Institute of Iran, Tehran, Iran; 4 Genetics, Genomics and Cancer Biology PhD Program, Thomas Jefferson University, Philadelphia, PA USA; 5 Department of Medical Genetics, School of Medicine, Tehran University of Medical Sciences, Tehran, Iran; 6 Skin Research Center, Shahid Beheshti University of Medical Sciences, Tehran, Iran; 7 Department of Dermatology, Children’s Medical Center, Center of Excellence, Tehran University of Medical Sciences, Tehran, Iran; 8 Cardiology Ward, Imam Sajjad Hospital, Mazandaran University of Medical Sciences, Mazandaran, Iran; 9 CMR Department, Rajaei Cardiovascular Medical and Research Center, Iran University of Medical Sciences, Tehran, Iran; 10 Viapath, St Thomas’ Hospital, London, UK; 11 Kawsar Human Genetics Research Center, Tehran, Iran; 12 Kariminejad-Najmabadi Pathology & Genetics Center, Tehran, Iran; 13 Division of Cardiology, Department of Medicine, Thomas Jefferson University Hospital, Philadelphia, PA USA; 14 St John’s Institute of Dermatology, King’s College London, Guy’s Campus, London, UK

**Keywords:** Molecular biology, Cardiology, Diseases

## Abstract

Arrhythmogenic right ventricular cardiomyopathy (ARVC), with skin manifestations, has been associated with mutations in *JUP* encoding plakoglobin. Genotype–phenotype correlations regarding the penetrance of cardiac involvement, and age of onset have not been well established. We examined a cohort of 362 families with skin fragility to screen for genetic mutations with next-generation sequencing-based methods. In two unrelated families, a previously unreported biallelic mutation, *JUP*: c.201delC; p.Ser68Alafs^*^92, was disclosed. The consequences of this mutation were determined by expression profiling both at tissue and ultrastructural levels, and the patients were evaluated by cardiac and cutaneous work-up. Whole-transcriptome sequencing by RNA-Seq revealed *JUP* as the most down-regulated gene among 21 skin fragility-associated genes. Immunofluorescence showed the lack of plakoglobin in the epidermis. Two probands, 2.5 and 22-year-old, with the same homozygous mutation, allowed us to study the cross-sectional progression of cardiac involvements in relation to age. The older patient had anterior T wave inversions, prolonged terminal activation duration (TAD), and RV enlargement by echocardiogram, and together with *JUP* mutation met definite ARVC diagnosis. The younger patient had no evidence of cardiac disease, but met possible ARVC diagnosis with one major criterion (the *JUP* mutation). In conclusion, we identified the same biallelic homozygous *JUP* mutation in two unrelated families with skin fragility, but cardiac findings highlighted age-dependent penetrance of ARVC. Thus, young, phenotypically normal patients with biallelic *JUP* mutations should be monitored for development of ARVC.

## Introduction

Arrhythmogenic right ventricular cardiomyopathy (ARVC), which constitutes up to 20% of sudden cardiac death cases in individuals under 30 years of age, is an inherited cardiac disease characterized by progressive fibrofatty replacement of right ventricular myocardium that increases the risk of ventricular tachycardia and early-onset sudden death^1^. This disease has an autosomal dominant mode of inheritance with considerably reduced penetrance with an estimated prevalence of 1:1000 to 1:5000 in the general population^2–4^. ARVC shows extensive genetic heterogeneity and has been previously associated with 14 different genes. The most common causal genes mutated in about 60% of ARVC patients include *PKP2*, *DSP*, *DSG2*, *DSC2*, and *JUP*, which all encode desmosomal protein components. Rare non-desmosomal variants in *TMEM43*, *TTN*, *TGFB3*, *RYR2*, *PLN*, *LMNA*, *DES*, and *CTNNA3* comprise less than 1% of all ARVC cases and have been reported in only a few families or clustering in specific areas, such as *TMEM43* in Newfoundland, Canada, and *PLN* in The Netherlands^5,6^.

While monoallelic mutations in desmosomal protein-encoding genes, such as *DSC2*, *DSP* and *JUP*, have been associated with isolated (non-syndromic) ARVC, biallelic mutations in these genes have been reported with concomitant phenotypes of ARVC and integumentary abnormalities, such as palmoplantar keratoderma (PPK) and woolly hair and/or alopecia^7–10^ (Table 1). The cardiocutaneous syndrome, Naxos disease, results from a two-base pair homozygous deletion in the plakoglobin gene, while its related variant, Carvajal syndrome, which shows greater left ventricular predominance, is due to a mutation in the desmoplakin gene. Furthermore, biallelic *JUP* mutations were reported in association with skin fragility in the spectrum of epidermolysis bullosa (EB), PPK, woolly hair and/or alopecia, and nail dystrophy. The prevalence of cardiac manifestations in these patients has remained uncertain because ARVC is a progressive disease that often does not become manifest until the 3rd or 4th decade of life. Patients with homozygous mutations, however, present earlier with 100% penetrance by adolescence^11^. Although the original report of biallelic *JUP* mutations indicated a complete penetrance in cardiac manifestations, the subsequent studies have reported variable outcomes: among seven patients reported in one study, only one patient progressed to left-sided dilated cardiomyopathy (DCM) by 19 years of age^11,12^; demise of two patients early after birth has been reported^13,14^; and several patients presented with neither ARVC nor any other cardiac abnormalities^15^. Therefore, genotype–phenotype correlations regarding the penetrance, age of onset, and variable phenotypes, specifically cardiac involvement in biallelic loss-of-function (LOF) *JUP* mutations has not been well established (Table 1).

**Table 1:** Summary of previously reported patients with biallelic homozygous *JUP* mutations presented with concomitant skin and cardiac manifestations.

Family No	No. of patients	Country of Origin	Age	Mutation	Exon/Intron	Integumentary manifestation	Cardiac involvement	Age at cardiac presentation
1	2	Kuwait^15^	6 m, 12 m	p.468G > A	Ex 3^#^	Skin fragility, PPK, sparse woolly hair, nail dystrophy	–	N/A
2	3^§^	Argentina^15^	2–11 y	p.Ser24^*^	Ex 2	Skin fragility, PPK, sparse woolly hair, nail dystrophy	–	N/A
3	1^§^	Argentina^12^	17 y	p.Ser24^*^	Ex 2	Skin fragility, PPK, sparse woolly hair, nail dystrophy	DCM (left)	19 y
4	1	N/R^14^	18 d^¶^	p.Gln184^*^	Ex 4	Skin fragility, alopecia, nail dystrophy	Perivascular fibrosis myocyte dropout**	N/A
5	1	N/R^13^	12 d^¶^	p.Gln539^*^	Ex 9	Skin fragility, alopecia, onycholysis	Unkown	N/A
6	2	Turkey^29^	34–46 y	p.Arg265His	Ex 5	NEPPK, alopecia	ARVC	20 y, N/R
7	7	France-Canada^30^	12–77 y	p.Glu301Gly	Ex 5	NEPPK, woolly hair	ARVC	18 y–48 y
8	19^+^	Greece^7^	N/R	p.Trp680Glyfs^*^11	Ex 12	NEPPK, woolly hair	ARVC	N/R
9	28^+^	Greece^11^	1–61 y	p.Trp680Glyfs^*^11	Ex 12	NEPPK, woolly hair	ARVC	12–68 y
10	2	Iran^(this study)^	2.5 y, 22 y	p.Ser68Alafs^*^92	Ex 2	Skin fragility, PPK, alopecia, nail dystrophy	Uncertain, ARVC	N/A

^§^, + : The patients are shared; ¶: deceased; d: day; m: month; y: year; N/R: Not reported; N/A: Not available; NEPPK: Non-epidermolytic palmoplantar keratoderma; ARVC: Arrhythmogenic right ventricular cardiomyopathy; DCM: Dilated cardiomyopathy.

^#^Synonymous mutation at the border of exon 3 and IVS 3 resulted in aberrant splicing.

**The results of post-mortem autopsy.

*JUP* encodes junction plakoglobin, also known as γ-catenin, an intracellular adapter protein that connects intermediate filaments to cadherins in desmosomes. Cell–cell adhesion complexes are abundant in tissues subjected to high levels of mechanical stress, such as the epidermis and the heart muscle^16^. In cardiomyocytes, plakoglobin plays a key role not only for anchorage of the myofibrillar apparatus to adherens junctions but also for myofibrillar compliance. Abnormal plakoglobin can cause desmosomal dysfunction, elevated degree of cardiac muscle stiffness, and increased total wall stress^17^.

As part of an effort to characterize a large cohort of patients with skin fragility disorders in the spectrum of EB patients in Iran, a country consisting of 80 million inhabitants with approximately 38% consanguineous marriages, patients with clinical presentations suggestive of different EB subtypes were subjected to genome-wide sequence analyses. Among the 362 genetically distinct families diagnosed with EB, two patients with a homozygous *JUP* mutation were found and are reported here.

## Methods

### Patient data

This study was approved by the Institutional Review Board of the Pasteur Institute of Iran. All subjects and the parents of child-subjects gave written informed consent to participate in this research and to allow their image to be published. All methods were performed in accordance with the relevant guidelines and regulations. A total of 362 patients from Iran were subjected to mutational analysis. The criterion for study inclusion was a tentative diagnosis of EB based on history of neonatal cutaneous blistering suggestive of a genetic skin fragility disorder^18–20^.

### Targeted next-generation sequencing panel

We developed a disease-specific, gene-targeted panel for next-generation sequencing to identify pathogenic variants in EB. This panel includes 21 distinct genes, 18 of which have been shown to harbor causative mutations in EB and three of which have been implicated in differential diagnosis of skin fragility disorders^18^. We tested this panel in a group of patients who are representative of a large multiethnic cohort of EB patients in Iran. Most patients had an undefined EB subtype, and in some cases only archived DNA was available. DNA was extracted from peripheral blood samples, taken from patients, their parents, and other clinically affected and unaffected family members, including siblings (if available), using a QIAamp DNA Blood Mini Kit (Qiagen, Valencia, CA) or the salting-out method. For DNA, target enrichment was performed using the TruSeq Custom Amplicon kit (Illumina Inc., San Diego, CA). DesignStudio (Illumina Inc.) was used for library design. All exons, at least 20 base pairs of the intron at each intron–exon boundary, and up to 50 base pairs of 5′- and 3′-UTRs were targeted. The designed library contained the genes *CD151, CDSN, CHST8, COL17A1, COL7A1, DSP, DST, EXPH5, FERMT1, ITGA3, ITGA6, ITGB4, JUP, KRT5, KRT14, LAMA3, LAMB3, LAMC2, PKP1, PLEC,* and *TGM5,* divided into 421 targets covered by 968 amplicon probes which were designed to cover 99% of the targeted bases. A total of 36,724,892 reads were aligned to the human genome, with the mean coverage of the target region being × 424^18^.

### Mutation analysis and transcriptome profiling of *JUP* gene expression by RNA-Seq

Total RNA was extracted from whole skin biopsy obtained from Patient 2 and from six healthy controls using TRIzol Reagent and quantified on a Nanodrop ND-100 spectrophotometer (Thermo Fisher Scientific, Wilmington, DE), followed by RNA quality assessment on an Agilent 2200 TapeStation (Agilent Technologies, Palo Alto, CA). Multiplexed library construction, workflow analysis, and sequencing runs were performed following standard Illumina protocols (Illumina, Inc., San Diego, CA) using the TruSeq Stranded Total RNA kit with indexes from Set A (Cat #: RS-122-2301). Paired-end (2 × 75) sequencing reads were generated on the Illumina NextSeq 500 platform and stored in FASTQ format. FASTQ quality was checked using FastQC, and the TruSeq RNA adapter sequences were removed by Trimmomatic. Alignment and mapping were performed using STAR-2pass (v. 2.5.3a) (https://github.com/alexdobin/STAR/releases) with the human reference genome (GRCh38/hg38) and GENCODE V27 annotations. In the first pass, an initial alignment was executed, and the splice junction information was collected. This information was then used for the second pass, in which the final alignment was performed. The alignments were sorted, the read group was added, and the corresponding index was created (using Picard tools). The static images of Sashimi plots were generated using the Integrative Genomic Viewer (https://software.broadinstitute.org/software/igv/. R programming language (https://www.r-project.org/) was used to perform the expression analysis and plotting to show the distribution range of expression values of several EB related genes and representative housekeeping genes in the patients versus the six controls^21,22^.

### Variant interpretation

After mapping the RNA-Seq results with human reference genome (GRCh38/hg38), the annotated CSV files of whole transcriptome sequencing from the proband was obtained for further analysis. To find the runs of homozygosity (ROH), we set a threshold of > 2 Mb and selected non-frameshift variants from the CSV file. Further, the sequence variants were filtered from the VCF files for missense, nonsense, and splice site-affecting variants. Indel variants were filtered for exonic in-frame insertions and deletions, frameshift mutations, and gained/lost start or stop codon. Additionally, only variants with total frequencies of < 0.001 or those without frequency data available in Genomes, gnomAD and ExAC databases were examined^23^.

### Homozygosity mapping

The single nucleotide polymorphism variants called from RNA-Seq data were used for homozygosity mapping by PLINK 1.90 beta (http://zzz.bwh.harvard.edu/plink/), and were then overlapped with ROHs in the patient’s genome. Considering the given phenotype, segregation analysis in the family, and the presence of several ROHs in the patient with consanguineous parents enabled us to identify a likely pathogenic homozygous variant in *JUP* in the proband^24^.

### Transmission electron microscopy

Skin biopsy specimens were cut into small pieces (< 1 mm^3^) and fixed in half-strength Karnovsky fixative for 4 h at room temperature. After being washed in 0.1 M phosphate buffer (pH 7.4), the samples were immersed in 1.3% aqueous osmium tetroxide (TAAB Laboratories, Berkshire, UK) for 2 h, incubated in 2% uranyl acetate (Bio-Rad, Hertfordshire, UK), dehydrated in a graded ethanol series, and then embedded in epoxy resin using propylene oxide (TAAB Laboratories). Ultra-thin sections were stained with uranyl acetate and lead citrate and examined in a Philips CM10 transmission electron microscope (Philips, Eindhoven, The Netherlands)^25^.

### Immunofluorescence microscopy

Skin sections (5 μm) from biopsy from traumatized area obtained from the patient 1 were air-dried and blocked with diluted normal goat serum (Sigma-Aldrich, Dorset, UK), and then incubated with mouse monoclonal anti-gamma Catenin antibody (Abcam, ab11506) diluted 1:250 in phosphate-buffered saline with 30% w/v bovine serum albumin (Sigma-Aldrich). At next step, after washing in phosphate-buffered saline, we labeled slides with fluorescein isothiocyanate secondary antibodies (Invitrogen, Paisley, UK). The sections were counterstained with DAPI. By excluding the primary antibody, negative controls were also generated for this study. We used the same camera and the exposure time (3 s) for image acquisition of all sections^26^.

## Results

### Case report

**Figure 1:**
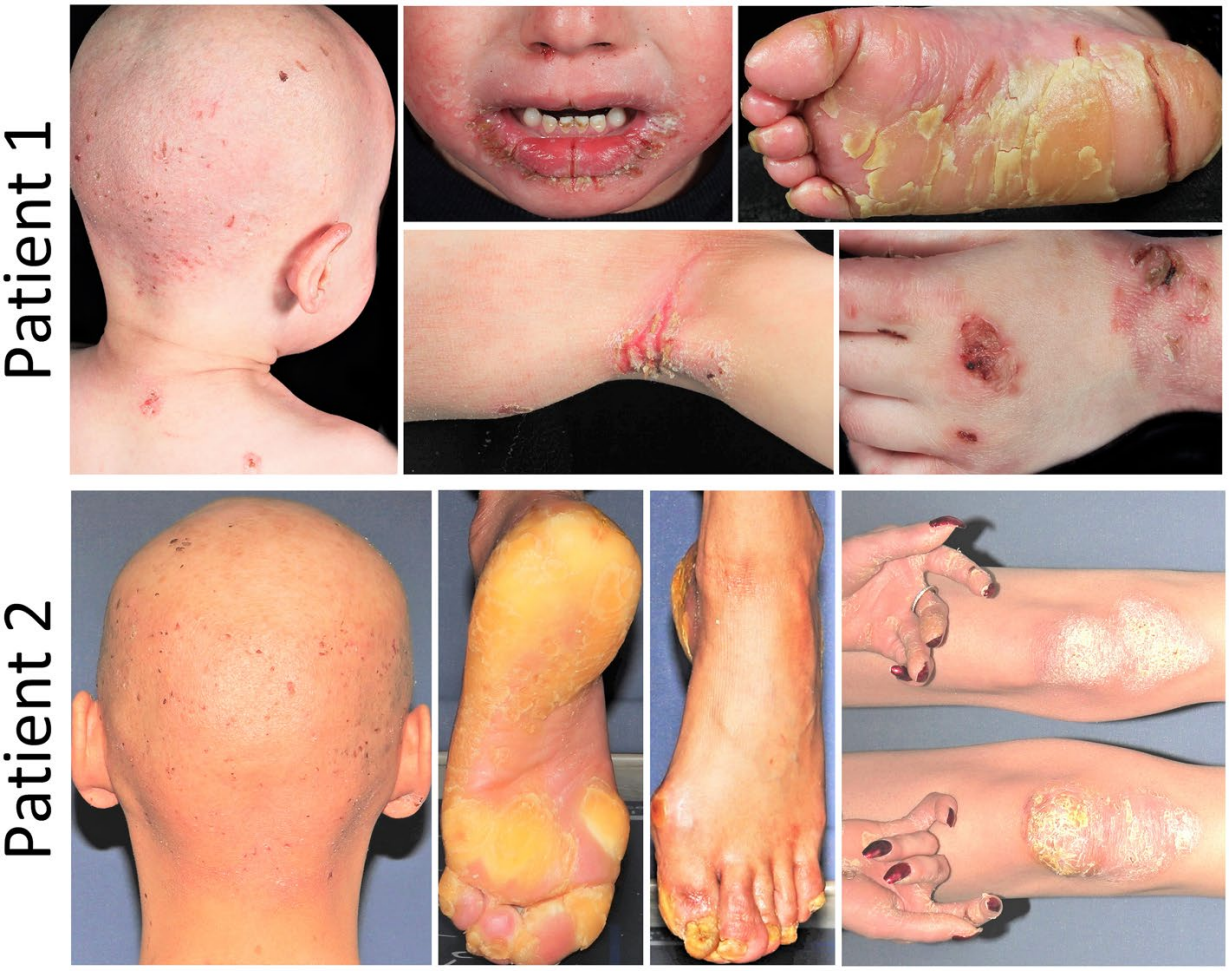
Clinical findings in patients with JUP-associated spectrum of skin fragility. Patient 1 (upper panel): A 2.5-year-old male manifested with trauma-induced blisters and erosions of the skin; perioral, axillary, plantar, and antecubital fissures; plantar hyperkeratosis; and absence of hair on his scalp. Patient 2 (lower panel): A 22-year-old female showed trauma-induced blisters and erosions; hyperkeratosis of palms, soles, knees and elbows; joint contractures of fingers; and absence of hair on her scalp.

Patient 1 was a 2.5-year-old male born at the 34th gestational week to healthy second cousin once removed consanguineous parents with Iranian-Azeri ancestry (Figs. 1 and 3a). He presented at birth with ulcers and erosions on the perioral and sacral areas. Several days after birth, an ulcer located posterior to the right internal malleolus developed (Fig. 1). At current examination, he presented with scattered erosions and ulcers of the skin, particularly in perioral and axillary areas, as well as plantar fissures; mucosal erosions; palmoplantar keratoderma (PPK); nail dystrophy proceeding to nail loss; and absent scalp hair, eyebrows, and eyelashes (Fig. 1). He exhibited failure to thrive and turned cyanotic while crying.

The laboratory tests revealed a normal serum zinc level. The electrocardiogram (EKG) and 24-h Holter monitoring showed sinus tachycardia with inverted T waves in right precordial leads (not shown). Transthoracic echocardiogram displayed normal ventricular ejection fraction, chamber size and motion. The parasternal long and short axis views of right ventricular outflow tract size (PLAX RVOT/BSA; PSAX RVOT/BSA) were normal (12.8 mm/m^2^ and 12.6 mm/m^2^, respectively). No cardiac abnormalities were found in the parents. The patient met one major criterion (biallelic *JUP* mutation) based upon the 2010 modified Task Force Criteria establishing a possible diagnosis of ARVC^27^.

Patient 2 was an Iranian-Azeri 22-year-old female born at the 33rd week of gestation to healthy second-cousin consanguineous parents (Fig. 3a). At birth, erosions on the scalp, alopecia, absence of eyebrows and eyelashes, dystrophic nails, and left-sided congenital clubfoot were noted. At current examination she presented with manifestations similar to Patient 1, including fragile skin; fissures at skin folds, periorificial areas, and the lateral canthus of the eyes; PPK; hyperkeratotic plaques and follicular hyperkeratosis on the knees and elbows; subungual hyperkeratosis; and nail dystrophy (Fig. 1). Alopecia, sparse curly eyebrows and eyelashes, short stature, low weight, and delay of puberty were also noted. Pruritus and skin fragility had lessened with advancing age. In the family, elective terminations of two subsequent pregnancies had been conducted.

**Figure 2:**
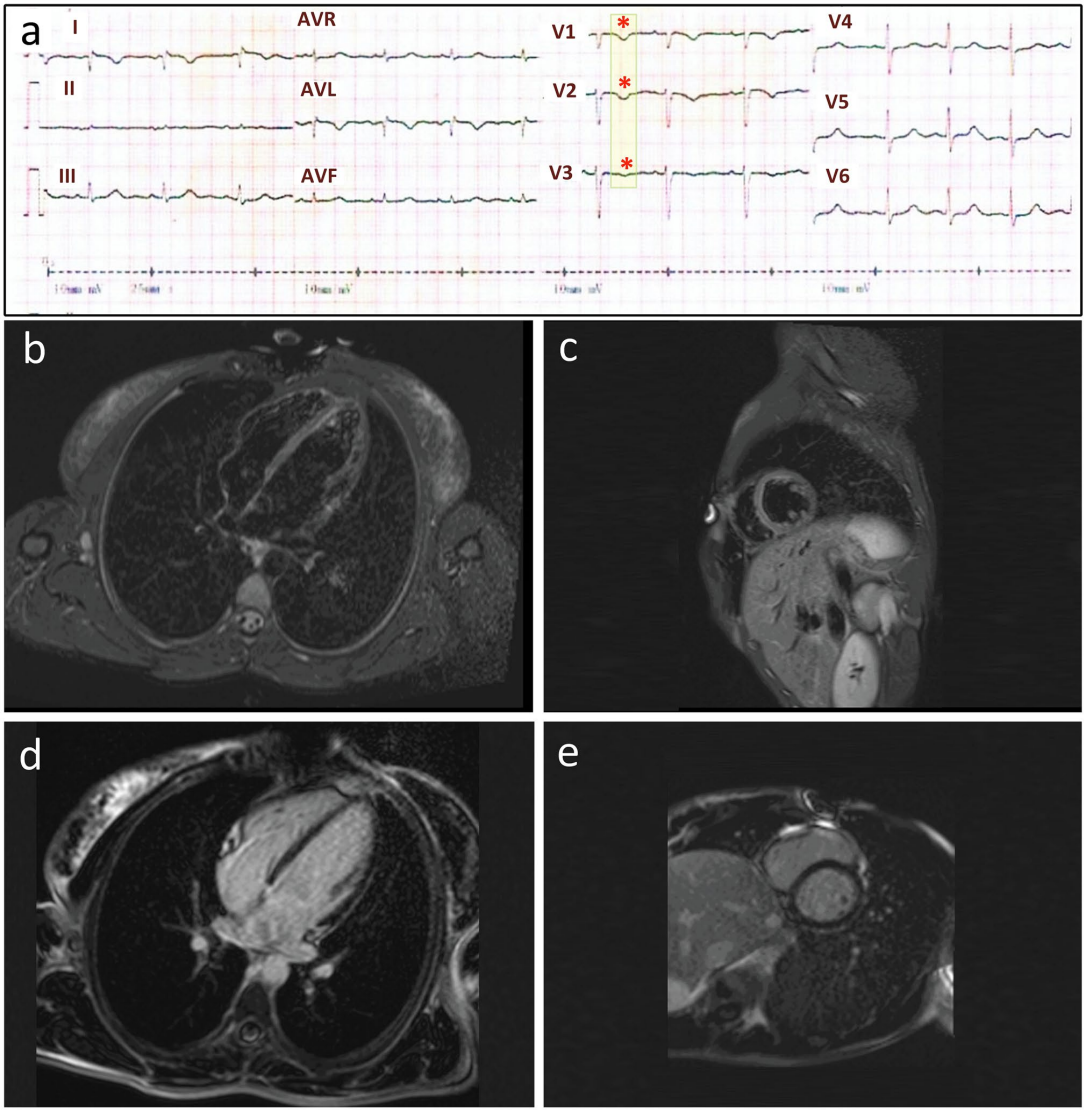
EKG, CMRI with Gadolinium findings in Patient 2. (**a**) EKG shows inverted T waves in leads V1, V2 and V3 (asterisks, major criterion) and a prolonged V3 terminal activation duration (TAD) = 80 ms (minor criterion). Epsilon waves are absent. (There is also a right arm/left arm limb lead reversal). CMRI shows normal myocardial thickness and wall motion along with normal left and right ventricular size, volume, and function (**b** and c). T2-weighted images (short-TI inversion recovery sequence) in the four-chamber (**b**) and short-axis (**c**) views of the ventricles show neither myocardial edema nor inflammation (**d** and **e**). Four-chamber (**d**) and short-axis views (**e**) of the ventricles in late Gadolinium enhancement sequences show no myocardial enhancement or evidence of myocardial replacement with fibrosis.

The EKG showed inverted T waves in right precordial leads (V1, V2, and V3) (Fig. 2a) and prolonged terminal activation duration in V1 (80 ms). A 24-h Holter monitoring showed no ventricular ectopy. Echocardiography revealed normal left ventricular ejection fraction (EF), but enlarged RV dimensions. The PLAX RVOT/BSA and PSAX RVOT/BSA were 21 mm/m^2^ and 24 mm/m^2^, respectively. Cardiac magnetic resonance imaging (CMRI), with and without Gadolinium, revealed normal myocardial thickness and wall motion, normal chamber size, volume and function, and no delayed myocardial enhancement (Fig. 2b–e). She met at least two major criteria (anterior T wave inversion and the *JUP* mutation) and one minor criterion (TAD > 55 ms) of the 2019 modified Task Force criteria giving her a definite diagnosis of ARVC^27^.

### Mutation detection

**Figure 3:**
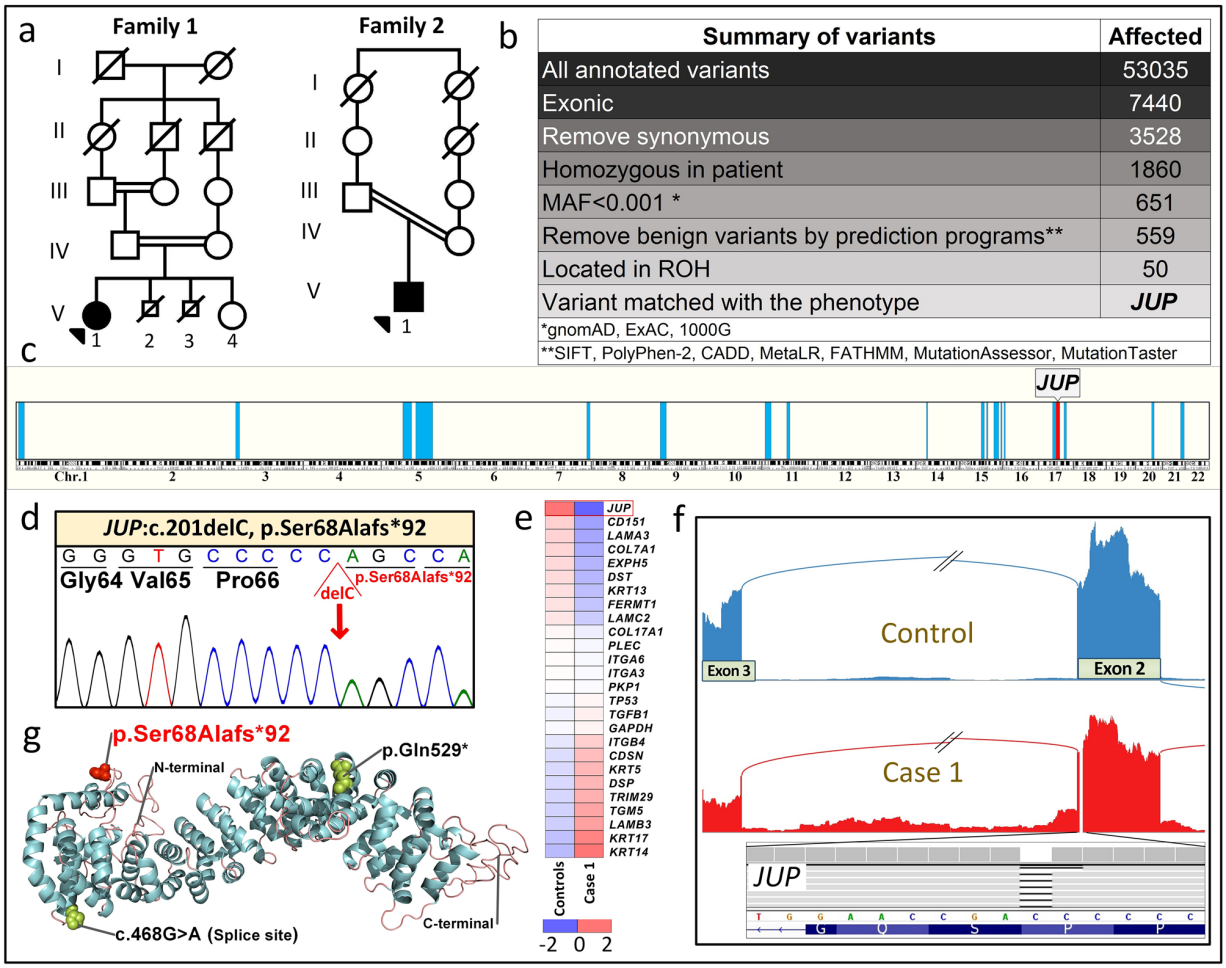
Identification of biallelic *JUP* mutations. (**a**) Pedigrees of two families with consanguinity. The proband in each family is indicated by an arrowhead. (**b**) Identification of the mutation in *JUP* by analysis of annotated variants from RNA-Seq data in Patient 2 using bioinformatics filtering steps indicated. (**c**) Homozygosity mapping based on RNA-Seq data localized the *JUP* gene within a homozygosity block of 10.7 Mb on chromosome 17. (**d**) Sanger sequencing confirmed the presence of the previously unreported homozygous mutation, *JUP*: NM_002230: exon 2, c.201_201delC, p.Ser68Alafs^*^92, in both probands. (**e**) Heatmap visualization of transcriptome analysis revealed markedly reduced level of *JUP* expression in comparison to the pool of six healthy controls when compared to other genes associated with EB phenotypes and randomly selected housekeeping genes. (**f**) Sashimi plot of the transcriptome profile of the mutant *JUP* mRNA revealed normal splicing, indicating lack of exon skipping and/or significant intron retention. (**g**) Protein visualization of full-length JUP consisting of 745 amino acids. The mutation identified in this study (red) is predicted to result in a frameshift and early truncation of the protein. Two previously reported *JUP* mutations in patients with similar phenotype are shown (black). The figure was rendered in PyMol (v.2, Schrodinger, New York, NY). For details of WES, homozygosity mapping, RNA sequencing and protein modeling, see PyMol, http://Rymol.org^18,24,28,43^.

Two different approaches were used for mutation detection. First, DNA-based next-generation sequencing (NGS) utilized a panel consisting of 21 genes associated with different skin fragility syndromes, including EB^18^. For targeted NGS, DNA was isolated from peripheral blood samples taken from the patients, their parents, and other clinically affected and unaffected family members. For details of the library construction, data capturing and bioinformatics, see the references^23,25^. The second mutation detection approach utilized RNA-based NGS via RNA-Seq analysis of a whole skin biopsy from Patient 2 as well as six unrelated healthy controls. The resulting data derived from transcriptome-wide variant calling (Fig. 3b), whole-genome homozygosity mapping (Fig. 3c), gene expression profiling visualized by heatmap (Fig. 3e), and gene-specific splicing events depicted by Sashimi plot (Fig. 3f), were elicited by using in-house bioinformatics algorithms^22^. Data analysis revealed the same homozygous mutation in *JUP*: [NM_002230], exon 2, c.201delC; p.Ser68Ala^*^92 in both probands, with their parents being heterozygous for the mutation. This previously unreported mutation was confirmed by bidirectional Sanger sequencing (Fig. 3d). This mutation is not reported in either homozygous or heterozygous state in gnomAD, GME Variome, ExAC, 1000 Genomes, or Iranome. The mutation was predicted to be pathogenic by Annovar, with a CADD score of 46. The 3D structure of junction plakoglobin protein is visualized by PyMol (v.2, Schrodringer, New York, NY), and previously reported mutations in patients without significant clinical cardiac involvement are shown in Fig. 3g^28^.

Since ARVC has also been associated with mutations in 13 other genes, besides *JUP*, including *PKP2, DSC2, DSG2, RYR2, SCN5A, TMEM43, DSP, CTNNA3*, *TGFB3*, *DES*, *LMNA*, *PLN* and *TTN*, the corresponding sequences were individually checked from the RNA-Seq data, and no pathogenic variants were found in these genes.

### Immunofluorescent staining

**Figure 4:**
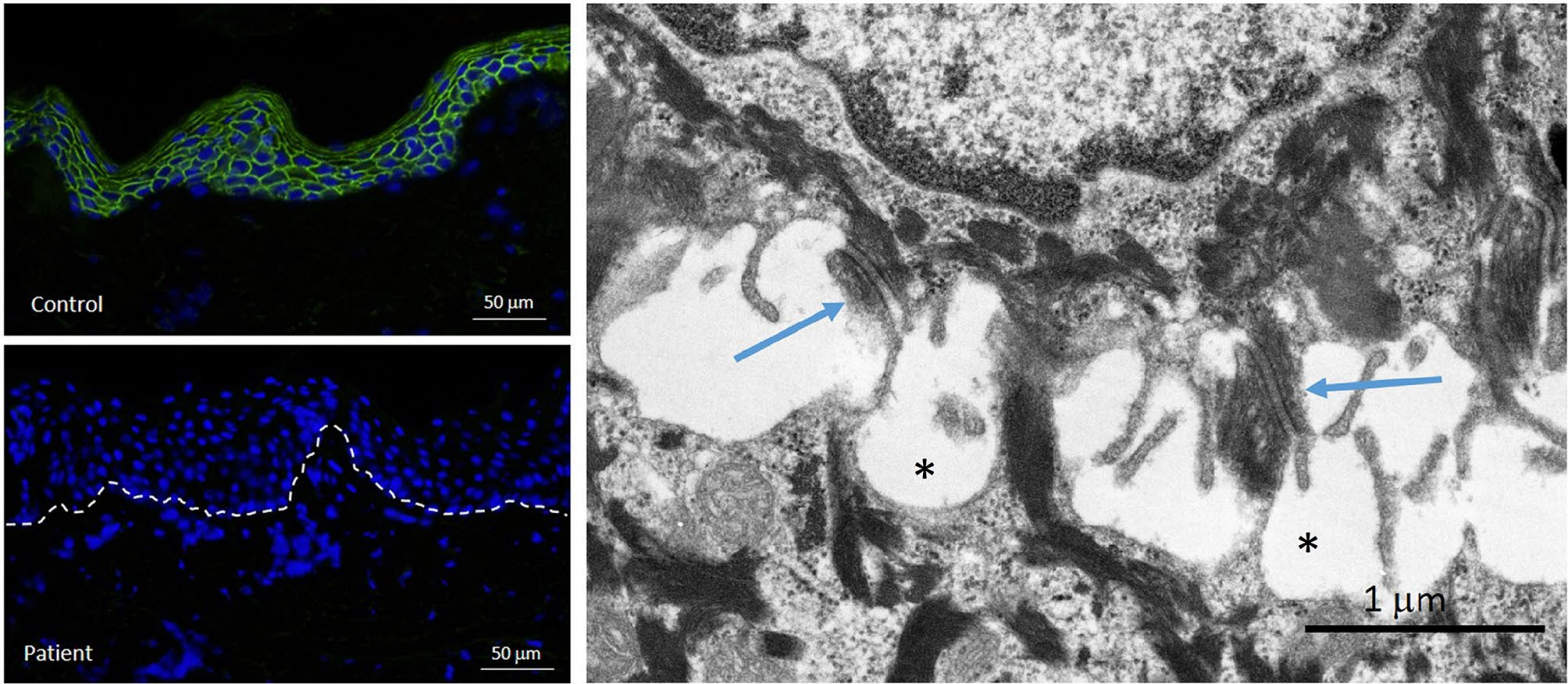
Immunofluorescence and transmission electron microscopy analysis of skin biopsy from Patient 2. IF staining for junction plakoglobin (encoded by *JUP*) shows complete absence of the protein in the epidermis of the patient (left lower panel) compared to pan-epidermal keratinocyte cell membrane labeling in an unrelated individual’s healthy skin (left upper panel). Ultrastructurally (right panel), desmosomes appear somewhat small with pinching off within the intercellular desmosomal plaques (arrows) and widening of the intercellular spaces between adjacent keratinocytes (asterisks). For technical details see reference^44^.

Immunofluorescence mapping using antibodies directed against desmosomal proteins (plakophilin-1, plakoglobin, and desmoplakin I/III) revealed essentially absent staining for plakoglobin in the epidermis of Patient 2 in contrast to pan-epidermal labeling at the periphery of keratinocytes in control skin (Fig. 4).

### Electron microscopy

Transmission electron microscopy of the skin in Patient 2 revealed intraepidermal blistering and keratinocyte lysis adjacent to desmosomal plaques (Fig. 4). Since the tissue separation is intraepidermal, these findings place these patients into the simplex category of EB.

## Discussion

In this study, we reported two patients from unrelated families residing in different parts of Iran harboring a homozygous mutation in the *JUP* gene*.* They both presented with skin fragility, PPK, alopecia and nail dystrophy, but ECG abnormalities were present only in the older patient. Neither had significant symptoms or family history for arrhythmia or heart failure. The significant age difference between the two patients provided us the opportunity to follow the progression of cardiac involvement in patients with the same biallelic mutation in *JUP* with time.

Literature review suggested full penetrance (100%) of ARVC in patients with biallelic *JUP* mutations, specifically p.Arg265His, p.Glu301Gly, p.Trp680Glyfs^*^11, and c.2157_2158delTG, presenting with PPK and woolly hair (Table 1)^7,11,29,30^. However, there was some degree of uncertainty about the cardiac involvement of the patients with biallelic *JUP* mutations presenting with skin fragility in addition to PPK and woolly hair/alopecia^12–15^. A biallelic *JUP* mutation that resulted in p.Ser24^*^ was primarily associated with skin fragility and ectodermal features without cardiac manifestations in three children from a single family (no. 2 in Table 1)^15^, although the eldest one manifested with left dilated cardiomyopathy (DCM) at 19 years of age^12^. However, it was not mentioned whether this patient was screened for mutations in any other ARVC/ALVC or DCM-associated genes. Moreover, DCM can be caused idiopathically or by other causes, such as viral infections. Another biallelic *JUP* mutation, c.468G > A, a synonymous splice site mutation at the exon3/intron 3 border, was associated with skin fragility, sparse woolly hair, and palmoplantar keratoderma, but no cardiac manifestations were noted in this family with two toddler siblings (Family 1, Table 1)^15^. Furthermore, biallelic mutations in *JUP*, p.Gln184^*^ and p.Gln529^*^, were reported with skin fragility, alopecia, and nail dystrophy; the patients died at 18 and 11 days after birth, respectively^13,14^. The family of the patient harboring the p.Gln184^*^ mutation consented to post-mortem autopsy, which revealed interventricular septal defect, patchy perivascular fibrosis, and myocyte dropout^14^. Review of the literature suggested that the nonsense and splice site mutations, located in more proximal exons of junction plakoglobin, including exons 2, 3, 4 and 9, manifested with more severe skin fragility as compared to the frameshift mutations leading to truncated protein in the distal exons, such as exon 12, or missense mutations in the exons 5 that were not associated with skin fragility (Table 1).

Cardiomyopathy in patients with biallelic *JUP* mutations, characterized by fibrofatty replacement of myocardium with subsequent ventricular dilatation and systolic dysfunction, carries a poor prognosis due to increased risk of ventricular arrhythmias and sudden cardiac death although the patients are often asymptomatic at an early age. Efficient screening of the patients could prevent the catastrophic complications later in life. The current recommended diagnostic approach for ARVC is non-invasive EKG monitoring and cardiac imaging, including echocardiography and CMRI^2,31^. Since most clinical findings alone are neither sensitive nor specific enough, 2010 Task Force criteria were developed to provide guidance on ARVC diagnosis^27^. Arrhythmogenic right ventricular cardiomyopathy is now considered within the spectrum of arrhythmogenic cardiomyopathies for which clinically relevant and updated information on genetics and disease mechanism are provided in a 2019 expert consensus statement^32,33^. The ARVC criteria are composed of six domains including global or regional dysfunction and structural alteration, histological characterization, repolarization abnormalities, depolarization/conduction abnormalities, arrhythmias, and family history^27^.

In the study by Groeneweg et al*.*, anterior (V1-V3) T wave inversion was found in 74% and prolonged TAD/epsilon waves were seen in 67% and 15% of patients, respectively, with a definite diagnosis of ARVC^34^. Inverted T waves in right precordial leads in an individual beyond 14 years of age, in the absence of right bundle branch block (RBBB), are found only in 4% of healthy women and 1% of healthy men, thus making it a major criterion with 47% sensitivity and 96% specificity for ARVC^35^. Furthermore, the pathogenic mutation in *JUP* is another major criterion, which counts for 0.5 to 2 percent of ARVC patients^36^.

The reason for the enlarged RV on ECHO but not CMRI in Patient 2 is unclear. However, because echocardiographic imaging of the RV can be limited, we based the absence of structural abnormalities on her CMRI. CMRI plays a major role in the diagnosis of ARVC in early stages. The sensitivity of CMRI is 79–89% for major and 68–78% for minor ARVC criteria while retaining > 95% specificity^37^. CMRI has some limitations including inter-observer variability in identifying features of ARVC, and it is therefore recommended that CMRI is assessed by an expert specialist familiar with this technique^37^. Moreover, the sensitivity of CMRI in children is low because arrhythmic presentations precede structural changes^38^. High negative predictive value of Gadolinium-enhanced CMRI makes it a good choice as a rule-out imaging test for evaluation of structural and functional abnormalities in patients suspected to have ARVC^39^.

*DSP* and *JUP* mutations accounted for 90.8% of the patients displaying cutaneous phenotype with ARVC^40^. Furthermore, most patients harboring a mutation in ARVC-associated genes present with ARVC by 30 years of age although homozygous patients (*e.g*., Naxos disease) show 100% penetrance by adolescence^42^. Long-term cardiac monitoring by EKG, Holter monitoring, echocardiography, and exercise stress test is recommended for the patients harboring mutations in ARVC-associated genes as well as those presenting with PPK and hair shaft anomalies with unknown genotype; however, it was reported that only 2.3% of patients with such cutaneous phenotypes receive relevant cardiac monitoring^41,42^. In this context, because of age-dependent penetrance and the young age of our patients, further studies and close follow-up are needed, including cardiological monitoring of family members at risk. Such risk can be assessed by mutation analysis of the members of the extended family.

In conclusion, we examined two unrelated patients with skin fragility, separated in age by 20 years, but who harbor the same, previously unreported homozygous *JUP* mutation. Repolarization/depolarization abnormalities and echocardiographic RV enlargement was seen in the older (definite ARVC) but not the younger (possible ARVC) proband. This cross-sectional observation highlights the age-dependent penetrance of ARVC, the need for close monitoring of patients with pathogenetic *JUP* mutations; and cascade screening of first degree at risk family members.

## Abbreviations

ALVC: Arrhythmogenic left ventricular cardiomyopathy
ARVC: Arrhythmogenic right ventricular cardiomyopathy
CMRI: Cardiac magnetic resonance imaging
DCM: Dilated cardiomyopathy
TAD: Terminal activation duration
EB: Epidermolysis bullosa
LOF: Loss-of-function
NGS: Next generation sequencing
PPK: Palmoplantar keratoderma
ROH: Runs of homozygosity
